# Identifying Salient Beliefs Underlying Young Adults’ Flavored E-Cigarette Use to Inform Campaign Development: Results from an Elicitation Survey

**DOI:** 10.1093/ntr/ntaf207

**Published:** 2025-10-13

**Authors:** Rebekah Wicke, Allison Worsdale, Jiaying Liu

**Affiliations:** Department of Communication, Cornell University, Ithaca, NY, United States; Department of Family Medicine, University of North Carolina at Chapel Hill, Chapel Hill, NC, United States; Lineberger Comprehensive Cancer Center, University of North Carolina at Chapel Hill, Chapel Hill, NC, United States; Department of Communication, University of California, Santa Barbara, Santa Barbara, CA, United States

## Abstract

**Introduction:**

The tobacco industry’s marketing efforts have successfully recruited a new generation of tobacco users, with flavored e-cigarette use becoming increasingly prevalent among young adults (YAs). Media campaigns continue to be an effective and cost-efficient approach to counteract the strong negative influence of marketing. Identifying salient beliefs underlying YAs’ flavored e-cigarette use serves as the first step in designing effective anti-vaping campaigns.

**Methods:**

An elicitation survey was conducted among 396 YAs (18–25 years; *M* = 19.66, *SD* = 1.45) who have ever vaped, with the majority (70.3%) having vaped in the past 6 months. Open-ended questions were used to probe perceptions of flavored e-cigarette use, including benefits, consequences, social norms, facilitators, and barriers of continued use. Thematic analysis was conducted by two coders (*α* = 0.81–1.00) to identify commonly held and novel beliefs.

**Results:**

The most shared benefits of flavored e-cigarette use included facilitating stress relief and addressing mental health concerns. While participants shared many health-related disadvantages of flavored vaping, including shortness of breath and addiction, our findings illuminate misperceptions and knowledge gaps about the safety of these products. Participants emphasized the influence of marketing on their use of flavored e-cigarettes, as appealing ads on social media were commonly cited as facilitating factors. Our findings also underscore the glorious social perceptions of flavored vaping.

**Conclusions:**

The findings of this study provide a comprehensive list of potential themes for anti-vaping campaigns targeting flavored e-cigarette use among YAs, ranked by prevalence. Novel, salient beliefs generated directly from the target audience provide fresh, innovative angles to fuel prevention efforts.

**Implications:**

This study fills an important gap in understanding about YAs’ beliefs and perceptions of flavored vaping products. Our findings reveal salient and novel attitudinal, normative, and control beliefs about flavored vaping. These beliefs will serve as a springboard for inspiring effective campaigns that seek to change YAs’ beliefs and to subsequently curb their flavored vaping behavior.

## Introduction

E-cigarette use has become increasingly prevalent among young adults (YAs) in the United States, including those who have never used traditional tobacco products (eg, cigarettes).^1^^,^^2^ The majority (89.6%) of YAs who vape use flavored e-cigarettes.^2^ This trend raises concerns about flavored vapes and their potential impact on the progress made in reducing tobacco use among YAs and youth.^3^ Flavored vaping products, commonly containing nicotine, not only possess addictive properties but also mask the unpleasant taste of nicotine with appealing flavors, making them more palatable for inhalation.^4^^,^^5^ Additionally, flavored e-cigarettes are often considered a gateway to broader nicotine consumption through other tobacco products,^6^ necessitating timely intervention. Despite regulatory efforts to restrict the sale of flavored e-cigarettes^7^ and proposals to ban menthol-flavored e-cigarettes,^8^ a substantial proportion of those who vape express intentions to continue using these products illicitly if they are banned.^9^ This underscores the need for more effective persuasive strategies to prevent initiation and encourage cessation of flavored vaping in this demographic.

Mass-mediated campaigns have long been utilized for health education and behavioral modification.^10^ However, recent systematic reviews highlight the lack of research with target audiences to inform campaign development.^11^^,^^12^ Engaging in research with the target population early is critical for identifying prevalent beliefs and perceptions, thereby enhancing the efficacy of subsequent campaigns.^13^^,^^14^ In light of this, our study applies the reasoned action approach (RAA) to lay the groundwork for crafting mass-mediated campaigns targeting flavored vaping among YAs in the United States. The RAA offers a structured, foundational framework for identifying the attitudinal, normative, and control beliefs that underlie individuals’ behaviors, guiding the classification and extraction of belief categories.^15^ While previous investigations have explored beliefs surrounding vaping, many have primarily focused on youth vaping or vaping in general, overlooking the nuanced realm of flavored vaping.^16^^,^^17^ Guided by the RAA, our study aims to identify YAs’ beliefs about flavored vaping to inform campaign development. This study represents the first phase of a broader research program aimed at testing the efficacy of messages that target prevalent and novel beliefs that YAs hold about flavored vaping, through experimental, psychophysiological, and intervention methods.

### The Reasoned Action Approach

The RAA serves as a robust framework in health communication scholarship, offering insightful pathways to understanding and altering YAs’ behaviors, particularly in the context of flavored vaping. Widely adopted by scholars, the RAA delves into the antecedents of behavior enactment, emphasizing the significant role of individuals’ beliefs in shaping their actions.^15^^,^^18^ This approach contends that an individual’s intention to engage in a behavior ultimately dictates their actions.^15^ Central to this intention are the individual’s underlying beliefs, categorized into three key domains: attitudinal, normative, and control.

Attitudinal beliefs reflect perceived advantages and disadvantages associated with the behavior of interest, offering insights into individuals’ motivations and concerns. Normative beliefs capture perceived social norms, including descriptive norms (what others do) and injunctive norms (what others expect one to do). Control beliefs address perceived barriers and facilitators, revealing factors that may enable or hinder behavioral performance. RAA asserts that to catalyze behavioral change, it is imperative to target and modify individuals’ beliefs effectively.^15^ Health campaigns tailored to YAs leveraging the RAA have demonstrated notable success across various domains, ranging from promoting dental check-ups to curbing drunk driving incidents.^19^^,^^20^ However, despite being the focus of public health campaigns addressing substance use, YAs are often excluded from the campaign development process, hindering the potential effectiveness of these interventions.^21^

**Figure 1:**
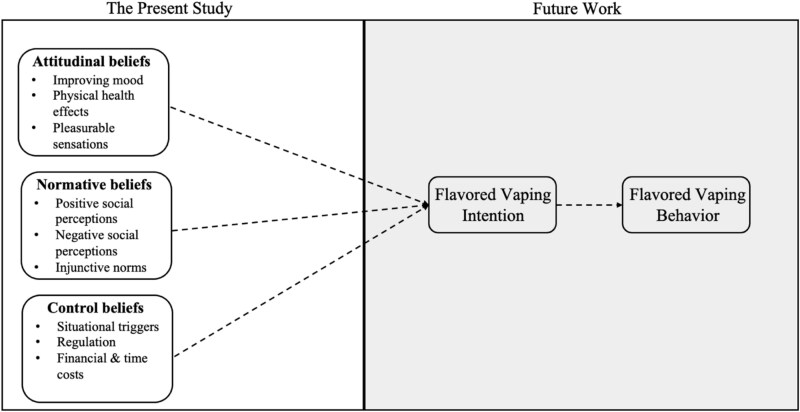
Elicitation of beliefs guided by the reasoned action approach. The figure above, adapted from past work,^43^ summarizes the reasoned action approach, highlighting the present study as the first step in identifying young adults’ (YAs’) attitudinal, normative, and control beliefs. Future work will test how messages aimed to target these beliefs ultimately influence YAs’ flavored vaping intentions and behavior.

One distinct advantage of the RAA lies in its provision of a theory-driven framework for pinpointing the most salient beliefs held by the target population. By identifying and understanding the prevalent beliefs and themes that necessitate modification or reinforcement, researchers and health practitioners can craft campaigns that resonate deeply with the target audience, thereby enhancing their efficacy and impact. Figure 1 summarizes the reasoned action framework guiding our study.

### Young Adults’ Beliefs Pertaining to Flavored Vaping

In recent years, combustible cigarette use among YAs has declined as public health efforts have taken hold.^22^ However, this positive trend has been offset by a sharp rise in vaping, especially among youth and YAs—a shift raising significant concerns due to both known and unknown health risks.^3^^,^^23^ The wide variety of flavored e-cigarettes, specifically designed to appeal to younger users, further complicates the issue.^5^ Although originally marketed as smoking cessation aids,^24^ e-cigarettes are often mistakenly perceived as safe alternatives to traditional cigarettes, undermining public health campaigns and highlighting the need to better understand YA perceptions of flavored vaping.^25^

Previous research has explored YA risk perceptions, finding that flavored e-cigarettes are commonly viewed as less harmful than tobacco-flavored e-cigarettes, largely due to their appealing flavors.^25^^,^^26^ Studies also reveal that social motivations and favorable attitudes toward flavored vaping shape usage, with participants citing benefits such as relaxation.^16^^,^^27^ Elicitation studies further demonstrate that beliefs about relaxation, mental health, flavors, and social perceptions strongly predict both vaping behavior and intentions, indicating that YA vaping is influenced by a combination of social factors and perceived health advantages.^28^

The prominence of flavored e-cigarettes highlights the importance of considering YAs’ attitudes and beliefs toward these products. Recognizing the role of flavors in shaping perceptions and preferences is essential to delve deeper into the motivations driving YAs to choose flavored e-cigarettes over combustible cigarettes or other tobacco products. Such insights are crucial for informing the development of targeted anti-vaping public health interventions tailored to address the specific beliefs and perceptions that drive YAs’ uptake of flavored e-cigarettes. Thus, the objective of this study is to identify YAs’ attitudinal, normative, and control beliefs about flavored vaping to inform campaign development.

## Methods

### Participants

Participants were recruited from a large Southeastern university. To participate, they needed to (1) be within the ages of 18 and 25 (ie, YAs) and (2) have used an e-cigarette in their lifetime. Participation in this study offered credit toward a research requirement for students.

### Study Protocol

The survey was distributed in an online format, and informed consent was obtained upon accessing the survey. Given that e-cigarettes are referred to by a variety of names, to ensure clarity, we first showed participants images of e-cigarette delivery devices and listed common names and slang terms used for e-cigarettes (eg, vapes, mods). We then asked participants to report their vaping habits, including the brands and flavors they use, as well as the frequency at which they vape. A series of open-ended questions followed, designed to elicit their attitudinal, normative, and control beliefs about flavored vaping. Lastly, participants provided basic demographic information. To help ensure that participants would provide responses pertaining to flavored e-cigarettes, rather than e-cigarettes more broadly, we reminded participants in each question that we were interested in their perspectives about flavored e-cigarettes specifically. This study was approved by the university’s Institutional Review Board.

### Instrument

Elicitation methodology broadly refers to the use of open-ended questions to uncover participants’ beliefs, attitudes, and perceptions surrounding a specific behavior. This approach is often used in research to inform the development of theory-driven campaigns.^29^ Guided by the RAA framework, elicitation surveys for health messaging campaigns typically aim to uncover three types of beliefs (ie, attitudinal, normative, and control beliefs) that serve as novel and salient targets for subsequent campaign message development.^30^^,^^31^ We developed our survey instrument by adapting established open-ended questions to the context of flavored vaping. Each participant was asked to provide up to five responses per open-ended question to ensure comprehensive exploration into their beliefs. Attitudinal beliefs were probed using questions such as, “*What are the [advantages/disadvantages] of using flavored e-cigarettes?*” These questions were aimed at identifying perceived benefits and consequences associated with flavored e-cigarette use.

To elicit normative beliefs, we began by identifying key social referents, that is, individuals or groups whose behaviors may influence participants’ vaping decisions, as understanding general normative beliefs alone may not yield actionable insights for campaign development. This approach enhances the utility of findings for designing messages that address social influences. Participants were queried to identify those who were most and least likely to engage in flavored e-cigarette use. Questions included, “*Please list all the individuals or groups who are [most/least] likely to use a flavored e-cigarette*.” Participants were also asked to describe a typical flavored e-cigarette user and reflect on the social implications of use, to surface how they believe the behavior is viewed by peers and society more broadly.

Control beliefs were explored through questions about situations that might lead someone to initiate or stop using flavored e-cigarettes. Questions included, “*What might prompt someone to [reach for/stop using] flavored e-cigarettes?*” These prompts aided in the identification of perceived facilitators and barriers to flavored vaping. Detailed question wording of belief-elicitation items is provided in the Supplement.

### Analysis

The open-ended data from this elicitation survey was coded by the first and second authors. We collaborated to develop a comprehensive and detailed codebook that guided the analysis. The codebook captured the belief categories informed by RAA (ie, attitudinal, normative, and control) and distinguished between pro-vaping (ie, advantages, facilitators, typical users) and anti-vaping beliefs (ie, consequences, barriers, and non-users) using a system of numbers (0, 1) and symbols (+/-). It is important to note that although the questions were designed to capture the three types of beliefs outlined by RAA, participants’ responses often crossed these categories. A single question could yield responses that aligned with different or multiple belief types, depending on how the participant interpreted and answered the prompt. For example, it was possible that participants noted normative or control beliefs when responding to questions designed to probe attitudinal beliefs. For responses containing multiple belief types, we broke down the responses into single entries and assigned a single belief label for each entry. Our codebook was developed to account for this. The development of the codebook was an iterative process, in which we revised the codebook to include and categorize various mentions of social referent groups and belief categories until it reached saturation. More information about the codebook can be found in the Supplement.

The two coders established high intercoder reliability ($\alpha =0.81-1.00$) using a subset of the data. The first author then coded all data in accordance with the codebook. The second author reviewed the coded data with the first author and assisted with determining how to code ambiguous responses. Beliefs were then categorized into overarching themes, and themes of similar focus were merged. Themes were ranked based on prevalence of occurrences.

## Results

### Sample Characteristics

The final sample consisted of 396 YAs who had ever vaped. Participants included 216 (54.5%) women and 177 men (44.7%); three participants did not indicate the gender they identify with. The average age of participants was 19.66 (*SD* = 1.45). Participants reported their racial backgrounds as white (77.1%), Asian/Pacific Islander (14.1%), Black or African American (4.3%), Hispanic or Latino/a (5.3%), Other (3.5%), and American Indian (1.0%). Most participants reported using e-cigarettes within the past 6 months (70.3%). Most participants reported using flavored e-cigarettes (90.0%).

### Salient Beliefs about Flavored Vaping

The most prevalent beliefs are summarized below, organized by belief type and by whether they supported or opposed flavored vaping. Percentages indicate the proportion each belief represents within its respective category.

#### Attitudinal Beliefs

*Advantages.*  Table 1 features salient attitudinal beliefs about flavored vaping. Participants generated 1287 advantage-related beliefs, which were grouped into 28 themes. The most frequently cited benefit was enjoyment and mood (ie, flavoring better facilitates stress relief; making up 33.1% of the advantage-related beliefs). Participants described using flavored e-cigarettes as a cost-effective and accessible means for managing stress. As one participant explained, “[*Flavored e-cigarettes] are available, quick stress reducers… It’s a whole lot easier than [paying] $125 an hour for therapy.”* This suggests that YAs are looking for solutions for their mental health concerns and thus may be motivated to use flavored e-cigarettes, as these products are perceived to be more affordable and just as effective as more conventional forms of mental health support (eg, seeing a counselor).

**Table 1:** Attitudinal Themes and Prevalence Ranking

Benefits		Consequences	
Improving mood (eg, stress relief)	33.1%	Physical health effects (eg, shortness of breath, coughing)	43.3%
Pleasurable sensations (eg, enjoyable tastes, smells)	31.9%	Addiction & bad habits	25.1%
Lower comparative risk to other substances	10.0%	Financial & time costs (ie, flavored vaping is an expensive & time-consuming hobby)	13.6%
Lower likelihood of addiction & bad habits (compared to other substances)	3.8%	Harmful ingredients	2.8%
Financial and time benefits (ie, flavored vaping is an affordable hobby)	2.9%	Lack of scientific evidence & knowledge about the harms of flavored vaping	2.2%
Anxiety management in lieu of prescription drugs	2.6%	Negative mood effects (eg, causing stress, depression)	2.0%
Ease of use	2.3%	Unpleasant sensations (eg, aftertastes)	1.8%
Appetite/weight management	2.1%	Gateway & poly use	1.7%
Cessation aid for traditional tobacco products	1.9%	Nicotine content	0.8%
Mental effects (eg, the light-headedness from flavored vaping)	1.6%	Unwanted appetite or weight loss	0.8%

The second most commonly shared benefit involved pleasurable sensations (eg, the various pleasing orosensory and olfactory stimulations generated by vaping flavors; 31.9%), with participants describing enjoying the appealing flavors and aromas of e-cigarettes. Participants also held the belief that a benefit of flavored e-cigarettes is the perceived lower risk compared to other substance use (10%). This perception was often tied directly to the presence of flavors. As one participant explained, “[*Flavored vaping] doesn’t seem as dangerous as smoking or doing drugs, especially when the flavor is something like candy or cereal.*” This belief highlights how sensory cues, like flavor, may reduce perceived harm and, in turn, influence motivation to try or continue using flavored e-cigarettes. These responses suggest that flavors not only enhance appeal but may also obscure users’ risk perceptions, which serve to reinforce the behavior.

*Disadvantages*. Participants generated 2354 beliefs about the disadvantages of flavored vaping. The most frequently noted disadvantages that may dissuade YAs from using flavored vapes included negative physical health effects (43.3%). Participants were aware of and concerned about a wide range of health effects associated with flavored vaping, including lung damage, mental illness, disrupted sleep, and nausea. Participants cited long-term health effects (eg, cancer, lung disease) more frequently (74.2% of negative physical health effects responses) than the immediate effects of flavored vaping (eg, headaches, vomiting; 25.7% of negative physical health effects responses). Other prevalent disadvantages included the loss of control caused by addiction (25.1%) and the financial/time costs associated with purchasing flavored e-liquids (13.6%).

#### Normative Beliefs

*Normative Referents and Social Perceptions*. Identifying salient normative referents, or social others, who would or would not engage in flavored vaping, serves as the first step of developing effective normative beliefs targeted by campaigns applying social norm appeals. Details about the prevalence of normative referents reported by the participants are summarized in Table 2.

**Table 2:** Normative Referents and Prevalence Ranking

Typical users		Nonusers	
Peers	48.3%	Parents	21.1%
Other	9.9%	Family (in general)	9.3%
Siblings	6.8%	Other	9.3%
High school students	5.5%	Peers	9.2%
Members of Greek life	5.5%	Grandparents	8.8%
Relatives	5.0%	Educators	7.5%
Coworkers	3.4%	Siblings	5.8%
Family (in general)	3.3%	Relatives	4.3%
Significant others	3.2%	Role models/authority figures	3.3%
Current smokers	2.1%	Kids/children	3.3%
Parents	1.5%	Older people/adults	3.0%
Athletes	1.1%	Health professionals	2.9%
Former smokers	0.9%	Elderly	2.8%
Older people/adults	0.8%	Religious individuals	2.2%
Kids/children	0.7%	Significant others	2.0%
Influencers/celebrities	0.6%	Athletes	1.6%
Educators	0.5%	Coworkers	1.0%
Role models/authority figures	0.4%	Current smokers	0.4%
Grandparents	0.2%	Members of Greek life	0.3%
Elderly	0.1%	Former smokers	0.3%
Religious individuals	0.1%	Influencers/celebrities	0.2%

*Positive Social Implications*. Participants also described the social benefits that motivate them to engage in flavored vaping with others, generating 1185 beliefs about the positive social implications of flavored vaping. They considered social bonding with and gaining respect from their friends who vape through sharing and offering flavored vapes as the leading social benefit (41.4%). As one participant explained, “*You can keep them on you and offer them to people for a hit, and it will make people think you’re generous and kind*.” In this sense, YAs may be motivated to use flavored e-cigarettes to derive social benefits and facilitate friendship development. Additionally, participants perceived being liked more when they used flavored vapes (36.2%) and considered it a prevalent behavior (19.9%; eg, “everyone does it”).

*Negative Social Implications*. Participants generated 577 beliefs about the social consequences of flavored vaping. Nearly half (48.5%) reflected concerns about being negatively perceived by others. Participants described fearing being perceived as unprofessional at job interviews, being judged by non-users as “lazy” or “addicted,” and being perceived as unattractive by potential romantic partners. Participants also described injunctive social norms (ie, disapproval from important others; 42.5%). These referents most frequently included parents and romantic partners, although many participants also cited concerns about disapproval from mentors, employers, and professors. A detailed breakdown of these social implications is provided in Table 3.

**Table 3:** Social Implications and Prevalence Ranking

Social benefits		Social consequences	
Social perceptions (positive)	41.4%	Social perceptions (negative)	48.5%
Injunctive norms	36.2%	Injunctive social norms	42.5%
Descriptive norms	19.9%	Harm to others	6.7%
Social anxiety management	1.4%	Situational triggers	1.0%
Celebrity influence	0.2%	Descriptive norms	0.5%
Lack of harm to others (compared to traditional tobacco products)	0.2%	Addiction & bad habits	0.3%
Curiosity & experimentation	0.1%	Mood effects	0.2%

#### Control Beliefs

*Facilitating Factors* **.** Salient control beliefs are reported in Table 4. Participants generated 3587 beliefs about the factors that facilitate flavored vaping. 47.0% mentioned situational triggers, including being in a social setting (eg, at parties, in bars), or in situations that elicit boredom (eg, being at home alone, driving) or stress (eg, doing schoolwork). Marketing influence, such as the appealing, vivid ad images of the flavored products, was the second most cited factor (15.3%). Participants also frequently noted enjoyment and mood (7.9%) as a motivating factor, with many describing vaping as relaxing and stress-relieving.

**Table 4:** Control Themes and Prevalence Ranking

Facilitators		Barriers	
Situational triggers (eg, settings like bars, parties; being stressed)	47.0%	Fear of physical health effects	35.3%
Marketing influence (ads, social media)	15.3%	Financial & time costs	15.8%
Enjoyment & mood	7.9%	Descriptive norms (hearing about others’ negative experiences)	11.2%
Being addicted to flavored vaping	5.3%	Scientific evidence & knowledge (ie, knowing more about the negative effects of flavored vaping)	10.0%
Gateway & poly use	4.7%	Addiction & bad habits	8.0%
Injunctive social norms (peer pressure)	4.4%	Self-image (eg, feeling badly about oneself for flavored vaping)	3.2%
Curiosity & experimentation	2.5%	Regulations	2.6%
Industry manipulation	2.1%	Situational triggers (eg, flavored vaping is inappropriate in certain settings or around certain people, like parents)	1.8%
Pleasurable sensations (eg, the alluring smell/flavors)	1.8%	Other	1.4%
Cessation aid for traditional tobacco products	1.4%	Anti-vaping messages	1.3%

*Barriers* **.** Participants generated 849 beliefs pertaining to the factors that motivate reduction or cessation of flavored vaping. Participants cited negative health effects (35.3% of barrier beliefs), such as decreased physical ability and developing popcorn lung. Experiencing shortness of breath was also frequently cited by participants. When describing what would prompt them to quit using e-cigarettes, one participant explained, “*Having difficulty breathing would be a major concern for me at a young age*,” suggesting that while YAs may initially underestimate the health risks associated with flavored vaping, experiencing shortness of breath would be alarming enough to motivate them to cease using these products.

Financial/time costs were among the most noted barriers to use, as participants described the mounting costs of maintaining their addiction as being unsustainable. A commonly shared belief was that if the prices of flavored e-cigarettes were significantly raised, YAs would no longer purchase them. In addition to financial costs, participants also described non-tangible costs, including the time that vaping takes out of their day, as well as the opportunity costs related to choosing flavored vaping over engaging in other hobbies and activities, as being potential barriers to continued use.

Hearing about others’ negative experiences (11.2%) was also a frequently cited barrier to use, with many participants explaining that others’ vaping “horror stories” and poor outcomes would motivate them to engage in cessation efforts. Notably, a non-trivial number of participants perceived there to be a limited amount of research about flavored vaping, noting that if there was scientific consensus that flavored vapes were harmful, they would be motivated to quit (10.0%). According to one participant, “*If information came out about the negative health aspects of flavored vapes, it could cause people to quit.*” This was echoed by other participants, including one who noted that researchers “*should announce an academic journal to show the real possible negative effects of flavored e-cigarettes*” to motivate YAs to quit. These findings suggest that some YAs’ continued use may be driven by uncertainty about harm, and that credible evidence may play a key role in shifting these beliefs.

## Discussion

Flavored e-cigarettes have surged in popularity since Juul’s 2015 debut,^32^ yet many health risks remain uncertain.^23^ These known and unknown risks highlight the need to intervene and reduce YAs’ flavored e-cigarette use. Public health campaigns have helped decrease YA tobacco smoking;^33^ similar efforts are now targeting vaping.^34^ This study aims to inform future campaigns by identifying the beliefs underlying YA flavored e-cigarette use, using RAA to assess attitudinal, control, and normative beliefs among those who have ever vaped. Our findings offer a ranked list of campaign themes and reveal novel beliefs directly from the target audience, providing fresh angles to counteract the appeal of flavored vaping.

Several studies, including our own, have delved into understanding YAs beliefs about vaping, contributing to a deeper comprehension of the factors influencing vape usage. Our findings resonate with those of similar studies, affirming established beliefs such as the perceptions of vaping as a means of gaining social acceptance and the belief that e-cigarettes pose less risk than traditional tobacco products (eg, cigarettes).^16^^,^^25^^,^^26^^,^^35^ Among the most prevalent attitudinal beliefs in our study was that flavored e-cigarettes are an effective means of stress management, which is in alignment with previous work that has found that YAs look to substances, such as e-cigarettes and cigarillos, to manage their mental health and regulate their mood.^17^^,^^28^^,^^36^^,^^37^ Consistent with past work on YAs’ beliefs about flavored vaping,^17^^,^^37^^,^^38^ the findings of our study also suggest that YAs recognize the addictive nature of flavored e-cigarettes and attribute their addictiveness to the appealing flavors.

Our findings also shed light on novel beliefs. Participants generated more control beliefs (*n* = 4436) than attitudinal (*n* = 3641) or normative beliefs (*n* = 1762), with most control beliefs focused on factors that facilitate use (*n* = 3587). Given that one strategy for designing effective campaigns is to target commonly held oppositional beliefs,^39^ messages addressing these facilitating factors may be particularly promising. For example, many participants cited boredom and stress as key drivers of use. Campaign designers may consider developing messages that offer alternative coping strategies such as hobbies, physical activity, or mindfulness practices. This aligns with prior critiques of anti-vaping PSAs, which often emphasize fear appeals related to health consequences but fall short in equipping audiences with actionable alternatives.^40^ Addressing control beliefs through empowering, solution-oriented messaging could therefore enhance message relevance and impact.

While it is possible that the prevalence of these beliefs is indicative of their importance in campaign design, it is also conceivable that this information has become saturated within the audience’s information environment, potentially diminishing its usefulness and novelty, and leading to message fatigue. Prominent campaigns, such as *The Real Cost* and the *Truth Initiative*, have long emphasized e-cigarette addictiveness and social drivers of use,^41^^,^^42^ which may explain their salience. Scholars have argued that messages that contain information that is well-known by a large portion of the intended audience may have limited capability when it comes to influencing the remaining portion of the audience; that is, if the information is convincing enough for much of the audience, the remaining portion of the audience may be particularly difficult to persuade.^39^ As such, future research should not discount the promise of less prevalent beliefs, as messages that are novel may be particularly effective with resistant populations.^44^

One such novel belief emerging in this study is that many YAs feel uncertain about the health risks of flavored vaping due to a perceived lack of scientific consensus. Several participants noted they would consider quitting if credible information about harms were made available. Although current campaigns often assert harm,^40^ few directly cite scientific evidence. Future work should test whether providing evidence-based messages that clearly communicate the degree of scientific certainty can meaningfully shift these beliefs and reduce flavored e-cigarette use. This finding also has practical implications for health and science communicators, underscoring the importance of disseminating research findings related to flavored vaping through channels that YAs frequent (eg, social media).

Although the purpose of this study was primarily to identify salient beliefs to inform health campaigns, our findings also have practical implications for policymakers. Notably, our participants emphasized cost as a primary barrier to use, with many noting that cost increases would be a deterrent for future flavored e-cigarette use. As such, regulators should consider raising the prices and taxes of flavored e-cigarettes to discourage YAs’ use.

## Limitations

This study used a convenience sample of college students. As such, our sample skewed younger (*M* = 19.66, *SD* = 1.45). Our results, therefore, may not be representative of all YAs who vape. However, considering that the college years are a time in which many young people initiate substance use,^45^ our results offer important insights for informing campaign efforts targeting college students who vape. Furthermore, our sample was predominantly white with higher-than-average levels of family income; our findings may have differed with a more diverse sample. Prior work has identified race as a predictor of vaping, finding that a high percentage of YA who vape identify as white^46^ and have high socioeconomic status,^47^ meaning that our sample demographics mirror those of typical YAs who vape. As past work has found that factors that influence vaping behavior, such as cultural norms and marketing exposure, can vary among different YA populations,^48^ future work should consider recruiting more diverse samples to explore how cultural and structural factors may shape attitudinal, normative, and control beliefs about flavored vaping.

Our sample also consisted of YAs who had ever used vapes and, therefore, contained both those who currently vape and those who previously vaped. Although many of our participants had vaped within the past 6 months (70.3%), a sample of only those who currently vape may have yielded different results. Segmentation analysis will be conducted for different subpopulations in our sample as the next step. Although our sample presented some limitations, it was also a strength of this study, as we had a substantially larger sample compared to many elicitation studies.^49^

## Conclusion

In conclusion, the findings of this study revealed YAs’ attitudinal, normative, and control beliefs about flavored vaping. These beliefs will serve as a springboard for inspiring effective campaigns that seek to change YAs’ beliefs to subsequently change their flavored vaping behavior. The next step in this research is to evaluate the efficacy of messages shaped by the salient and novel beliefs identified in this study, using hypothesis-driven, quantitative, and inferential methods.

## Supplementary Material

Young_Adults_Salient_Beliefs_NTR_Supplement_ntaf207

## Data Availability

Data is not publicly available to protect the anonymity of participants
