# Supplementary material for: Identifying Salient Beliefs Underlying Young Adults’ Flavored E-Cigarette Use to Inform Campaign Development: Results from an Elicitation Survey
Source: Nicotine Tob Res. 2025 Oct 13;28(4):554–62. doi: 10.1093/ntr/ntaf207 (PMC13008585; doi:10.1093/ntr/ntaf207)
Supplement: Young_Adults_Salient_Beliefs_NTR_Supplement_ntaf207 [file young_adults_salient_beliefs_ntr_supplement_ntaf207.docx]

**Supplemental Material**

Identifying Salient Beliefs Underlying Young Adults’ Flavored E-Cigarette Use to Inform Campaign Development: Results from an Elicitation Survey

Contents:

S1: Survey Questions

S2: Coding Approach

S1: Survey Questions

Questions Probing Attitudinal Beliefs

- What do you see as the **advantages** of using a flavored electronic cigarette? What do you **like** about it? Write each thought in a separate text box. We have prepared 5 text boxes below. If you come up with fewer than 5 advantages, put an “N/A” in the text boxes that you won’t be able to fill in. If you come up with more than 5 advantages, please continue to provide your thoughts in the last text box (i.e., the fifth text box).
- What do you see as the **disadvantages** of using a flavored electronic cigarette? What do you **dislike** about it? Write each thought in a separate text box. We have prepared 5 text boxes below. If you come up with fewer than 5 disadvantages, put an “N/A” in the text boxes that you won’t be able to fill in. If you come up with more than 5 disadvantages, please continue to provide your thoughts in the last text box (i.e., the fifth text box).

Questions Probing Normative Beliefs

- Sometimes when we are not sure what to do, we look to see what others are doing. Please list ALL the individuals or groups (no names, just type of relationships, such as friends, family members etc.) who are **MOST likely** to use a flavored electronic cigarette.
- Please list ALL the individuals or groups who are **LEAST likely** to use a flavored electronic cigarette.
- In a few words, describe the typical flavored e-cigarette user your age.
- What are the social implications of using flavored e-cigarettes?

Questions Probing Control Beliefs

- What might prompt someone to **reach for** a flavored e-cigarette? This could be a situation, a person, or a moment, etc. Please list ALL the occasions (e.g., places, events) in which one might be **inclined to use** flavored electronic cigarettes. Write each occasion on a separate text box. We have prepared 5 text boxes below. If you come up with fewer than 5 occasions, put an “N/A” in the text boxes that you won’t be able to fill in. If you come up with more than 5 occasions, please continue to provide your thoughts in the last text box (i.e., the fifth text box).
- What might prompt someone to **stop using** flavored e-cigarettes? This could be a situation, a person, or a moment, etc. Please list ALL the occasions (e.g., places, events) in which one might **find it hard to continue** using flavored electronic cigarettes. Write each occasion on a separate text box. We have prepared 5 text boxes below. If you come up with fewer than 5 occasions, put an “N/A” in the text boxes that you won’t be able to fill in. If you come up with more than 5 occasions, please continue to provide your thoughts in the last text box (i.e., the fifth text box).

S2: Coding Approach

Step-by-Step Codebook Instructions

Step 1: To prepare the data for coding, label each participant’s response with their participant ID. Then, copy their open-ended responses into a separate document in a format that allows easier line-by-line coding. Each response was transposed so that each comment appeared in its own row, with the corresponding participant ID clearly marked.

Step 2: After transferring the responses, remove any blank entries or responses marked “N/A” to ensure only meaningful data are coded. This process helps organize the data consistently while preserving participant identifiers for accurate thematic coding.

If a participant provides multiple responses in one entry (e.g., “Parents will be disappointed. Friends will think you’re cool.”), **insert a new row** below and separate each distinct statement so they can be coded individually. Even if the beliefs fall under the same theme, they should be coded separately if they express different ideas. The only exception is when a participant is telling a cohesive story and the combined statements clearly represent a single belief or theme.

*Examples:*

*[stop use] health consequences, parents find out, can’t afford it, if someone judges you = 4 beliefs*

*[prefer flavors] flavors make you seem like you aren’t doing anything bad and they’re enjoyable=2 beliefs*

*[social implications] you’ll fit in with other people who vape. You’ll be seen as cool. = 2 beliefs*

*[occasion] If your friends are vaping, it will make you want to vape too. You don’t want to be the uncool one who isn’t vaping. You’ll show them you’re a fun, cool person if you vape too. = 3 beliefs*

*[typical user] A college student, maybe someone who is rich, someone who doesn’t care what other people think of them and is very social = 4 beliefs*

*[nonuser] my mom and grandma = 2 beliefs*

*[advantage] helps you chill out, focus, and do better in school = 3 beliefs*

Step 3: Code each entry by belief type

*Note:* Every response (i.e., each entry) must be assigned a category.

There are three belief categories based on the Reasoned Action Approach:

**1. Attitudinal Beliefs**

These reflect **perceived benefits or consequences** of vaping flavored e-cigarettes.

*Examples:*

- *Flavored e-cigarettes help me relax.*
- *They taste good.*
- *Flavored vapes help me manage my appetite.*
- *They are unhealthy.*
- *They cause cancer.*
- *I get a headache after I vape.*
- *I don’t do well at sports when I vape.*
- *They give you a nice buzz.*

**2. Normative Beliefs**

These reflect perceptions of **what others do** (descriptive norms) and **what others approve or disapprove of** (injunctive norms) regarding flavored vaping. They may also include broader **social impressions**, such as beliefs about who uses e-cigarettes and perceived approval, disapproval, or expectations from peers, family, or society.

*Examples:*

- *Doctors would not use flavored vapes.*
- *Fraternity members would use flavored vapes.*
- *My parents wouldn’t like it if they knew I vaped.*
- *I will be popular if I vape.*
- *Vaping makes you look cool.*
- *People judge you if you use flavored vapes.*
- *You’ll fit in if you vape.*
- *If my friends offer me a flavored vape, I will use it.*
- *Social gatherings are ideal for vaping.*
- *People bond over using flavored vapes.*

**3. Control Beliefs**

These reflect factors that **enable/facilitate** or **hinder** vaping.

*Examples:*

- *Being drunk makes me vape more.*
- *I can’t afford my flavored vapes.*
- *If my vape pen breaks, I might consider quitting.*
- *Personal growth would make someone stop using flavored vapes.*
- *Having a bad experience with flavored vapes would make me stop.*
- *Wanting to try a new flavor*
- *Sometimes my cravings make me reach for a flavored vape.*
- *Boredom makes me reach for flavored vapes.*
- *Being at work is an occasion in which I would use flavored vapes.*

There is one column for each of the three belief types. Enter **1** in the column if the response reflects that belief type (e.g., attitudinal), and **0** if it does not.

For example:

| Original Response | Attitudinal | Normative | Control |
| --- | --- | --- | --- |
| Flavored vapes are harmful to my health. | 1 | 0 | 0 |
| Everyone else is vaping, so it is okay for me to vape too. | 0 | 1 | 0 |
| Sometimes I can’t afford my flavored vapes; they’re too expensive. | 0 | 0 | 1 |

Sometimes a response may seem to fit more than one belief category. In these cases, referring back to the original survey question can help clarify the best fit. If it still aligns with multiple categories after review, feel free to mark “1” in all relevant columns. We’ll review these entries later to determine the most appropriate classification.

**Note: Steps 4 - 5 apply specifically to extracting normative beliefs from the reference groups identified by participants.*

Step 4: For questions asking participants to list those most and least likely to use flavored e-cigarettes, convert the reference groups mentioned into belief statements.

Most responses to these questions are brief, one-word answers (e.g., mother, siblings, roommate) and should be translated into full belief statements. In the *User Beliefs* column in the coding spreadsheet, write a short statement that clearly reflects the implied belief.

For example, if a participant wrote “Professor” in response to the “least likely user” question, you may write: “My professors would not use flavored e-cigarettes.” Conversely, if they listed “friends” in response to the “most likely user” question, you could write: “My friends would use flavored vapes.”

Step 5: Label the reference groups mentioned using the coding scheme below:

1=Parents

2=Siblings

3=Grandparents

4=Other Relatives

5=Peers, Friends, Roommates, Acquaintances, College Students, Young adults

6=Educators (e.g, teachers, professors)

7=Health Professionals (e.g., doctors, nurses)

8=Authority Figures (e.g., boss, role model)

9=Elderly (excluding participant’s own grandparents)

10=Youth (e.g., high schoolers, teenagers)

11=Children/Kids

12=Individualsin Greek Life

13=General Family members (use only if participants wrote “family” or “family members”)

14=Influencers/Celebrities

15=Older Adults

16=Coworkers

17=Current Smokers

18=Former Smokers

19=Significant Other/Partner

20=Religious Individuals

21=Athletes

22=Other (please specify)

**Note: Step 6 applies specifically to extracting descriptive and injunctive norm beliefs from responses already identified as normative beliefs.*

Step 6: For each response identified as a normative belief, indicate the norm type (descriptive or injunctive) in the *Norm Type* column in the coding spreadsheet.

There are two types of normative beliefs related to reference groups:

Descriptive Normative Beliefs – who would or would not use flavored e-cigarettes

*Examples:*

- *My parents would not use flavored vapes.*
- *My friends would use flavored vapes.*

Injunctive Normative Beliefs – who would approve or disapprove of using flavored e-cigarettes

*Examples:*

- *My significant other wouldn’t like it if I used flavored vapes.*
- *My parents would be angry with me if they caught me using flavored e-cigarettes.*
- *My friends encourage me to vape.*

Coding Instructions:

- If the belief reflects a descriptive norm, enter 1 in the *Norm Type* column.
- If it reflects an injunctive norm, enter 2.
- If it’s a normative belief, but doesn’t clearly reflect either type, enter N/A.

Step 7: Valence coding for **all data** (in the “+/-” column):

*Note*: Every response (i.e., each entry) must be coded for valence (i.e., whether the belief supports or opposes flavored e-cigarette use).

Valence Coding Instructions:

Use a “+” sign for beliefs that support or encourage flavored e-cigarette use, and a “–” sign for beliefs that discourage or oppose use. Apply this across all belief types:

- Attitudinal Beliefs
- “+” for positive outcomes (e.g., “*Using flavored vapes helps me relieve stress.*”)
- “–” for negative outcomes (e.g., “*My lungs hurt when I use flavored vapes.*”)
- Normative Beliefs
- “+” if the belief reflects approval, use, or positive social implications (e.g., “*My peers would use flavored vapes.*” / “*I will look cool if I vape.*”)
- “–” if it reflects disapproval, nonuse, or negative social consequences (e.g., “*My parents would not use flavored vapes.*” / “*People will judge me if I vape.*”)
- Control Beliefs
  - “+” for enabling factors (e.g., “*My cravings make me want to vape*.” / “*Boredom drives me to use flavored e-cigarettes*.”)
  - “–” for barriers (e.g., “*I can’t afford flavored vapes.*” / “*Learning about the chemicals would make me stop.*”)

Step 8: Code the beliefs into themes:

*Note:* Every response (i.e., each entry) must be assigned a theme.

**Examples of common themes identified during codebook development:**

Below are example themes that have emerged during codebook development. As you code, feel free to add new themes if none of the existing ones apply. For any new theme, please document it with a brief explanation in a separate Word file and submit it with your coded data.

**Attitudinal Themes**

- Long-term health effects – concerns about long-term, chronic health consequences of vaping
- Immediate health effects – reflect more immediate, short-term symptoms (e.g., stomachache, headache, etc.)
- Addiction – beliefs about developing dependence on nicotine
- Stress relief – vaping as a way to relax or manage stress
- (*Add and label new themes as they emerge*)

**Normative Themes**

- Social acceptance – vaping helps fit in, look cool, be more approachable
- Normalized behavior – belief that “everyone” or “most people my age” vape
- Disapproval – concern that others (e.g., parents, authority figures) would disapprove
- Getting caught – fear of social consequences or needing to hide vaping
- Typical user – descriptions of who is likely to vape
- Nonuser – descriptions of who is unlikely to vape
- (*Add and label new themes as they emerge*)

**Control Themes**

- Addiction – vaping driven by cravings or inability to quit
- Appealing flavor/taste – flavor as a motivator or enabling factor
- Cost prohibitive – high cost as a barrier to vaping
- *(Add and label new themes as they emerge)*

Handling Cross-Category Responses

When coding, do not automatically assign a belief type based on the question it was responding to. Instead, code each belief based on its actual content.

Use the original survey question only to help contextualize ambiguous responses. For example, if a participant simply wrote “addiction,” check the question it was answering:

- If it was in response to a question about perceived disadvantages of flavored vaping, code it as an attitudinal belief.
- If it was in response to a question about what might lead someone to vape, code it as a control belief.

Participants may respond to a question designed to elicit one belief type with a belief that fits another category. This is common, especially with questions about perceived advantages and disadvantages, which may yield normative beliefs (e.g., social advantages and disadvantages) and control beliefs (e.g., addiction framed as a disadvantage, but also a barrier to quitting).

Refer to the definitions provided for attitudinal, normative, and control beliefs (based on the Reasoned Action Approach) to determine the best fit. Always prioritize the meaning of the belief itself over the intention behind the question prompt.

For example, when coding beliefs about addiction in response to the question “*What do you see as the disadvantages of using a flavored electronic cigarette?*” (intended to elicit attitudinal beliefs), determine whether the response reflects a negative consequence of use (attitudinal), social implications of use (normative), or a barrier to quitting (control).

| Original Response | Attitudinal | Normative | Control |
| --- | --- | --- | --- |
| I hate my addiction, but I don’t know how to stop. | 0 | 0 | 1 |
| You’ll lose your friends if you get addicted. | 0 | 1 | 0 |
| There’s a chance you might develop a dependence. | 1 | 0 | 0 |
